# A herbal medicine for Alzheimer’s disease and its active constituents promote neural progenitor proliferation

**DOI:** 10.1111/acel.12356

**Published:** 2015-05-25

**Authors:** Jianxin Mao, Shichao Huang, Shangfeng Liu, Xiao-Lin Feng, Miao Yu, Junjun Liu, Yi Eve Sun, Guoliang Chen, Yang Yu, Jian Zhao, Gang Pei

**Affiliations:** 1 State Key Laboratory of Cell Biology, Institute of Biochemistry and Cell Biology, Shanghai Institutes for Biological Sciences, Chinese Academy of Sciences320 Yueyang Road, Shanghai, 200031, China; 2 Graduate School, University of Chinese Academy of Sciences, Chinese Academy of Sciences320 Yueyang Road, Shanghai, 200031, China; 3 Department of Ophthalmology, Shanghai Tenth People’s Hospital, Tongji University School of MedicineShanghai, 200072, China; 4 Institute of Traditional Chinese Medicine and Natural Products, College of Pharmacy, Jinan UniversityGuangzhou, 510632, China; 5 Key Laboratory of Structure-Based Drug Design & Discovery, Ministry of Education, Shenyang Pharmaceutical UniversityShenyang, 110016, China; 6 Translational Center for Stem Cell Research, Tongji Hospital, Tongji University School of MedicineShanghai, 200065, China; 7 School of Life Science and Technology, and the Collaborative Innovation Center for Brain Science, Tongji UniversityShanghai, 200092, China

**Keywords:** asarone, ERK cascade, neural progenitor cell proliferation, neurodegeneration, traditional herb medicines

## Abstract

Aberrant neural progenitor cell (NPC) proliferation and self-renewal have been linked to age-related neurodegeneration and neurodegenerative disorders including Alzheimer’s disease (AD). Rhizoma *Acori tatarinowii* is a traditional Chinese herbal medicine against cognitive decline. In this study, we found that the extract of Rhizoma *Acori tatarinowii* (AT) and its active constituents, asarones, promote NPC proliferation. Oral administration of AT enhanced NPC proliferation and neurogenesis in the hippocampi of adult and aged mice as well as that of transgenic AD model mice. AT and its fractions also enhanced the proliferation of NPCs cultured *in vitro*. Further analysis identified α-asarone and β-asarone as the two active constituents of AT in promoting neurogenesis. Our mechanistic study revealed that AT and asarones activated extracellular signal-regulated kinase (ERK) but not Akt, two critical kinase cascades for neurogenesis. Consistently, the inhibition of ERK activities effectively blocked the enhancement of NPC proliferation by AT or asarones. Our findings suggest that AT and asarones, which can be orally administrated, could serve as preventive and regenerative therapeutic agents to promote neurogenesis against age-related neurodegeneration and neurodegenerative disorders.

## Introduction

Proliferation and self-renewal of neural progenitor cells (NPCs) persist throughout life in mammal brains and are critical for neurogenesis (Zhao *et al*., 2008). Decreased NPC proliferation and self-renewal occur under conditions such as aging, chronic stress, and central nervous system disorders and thereby may contribute to cognitive impairment (Haughey *et al*., 2002; Donovan *et al*., 2006; Drapeau *et al*., 2003; Drapeau & Nora Abrous, 2008; Seib *et al*., 2013). Promoting neurogenesis has been considered as a potential preventive and therapeutic strategy for anti-aging and neurodegenerative disorders such as Alzheimer’s disease (AD) (Lie *et al*., 2004). Further, transplantation of fetal NPCs or NPCs induced *in vitro* by defined factors has been shown to improve learning and memory in mice (Fan *et al*., 2014). However, there are still some open questions coming along with this technique such as donor cell source and functional integration. On the other hand, mobilizing endogenous NPCs by pharmacological agents should provide an alternative cell therapy for neurodegeneration (Miller & Kaplan, 2012). Considering that pharmacological agents can be delivered easily and can target certain aspects of NPC function, mobilizing endogenous NPCs should not only be considered as a feasible therapeutic approach but also be a preventive strategy (Jin *et al*., 2006; Fiorentini *et al*., 2010; Wang *et al*., 2010, 2012).

Neural progenitor cell proliferation is regulated by a variety of extracellular factors and intracellular pathways. Upon stimulation of the growth factors, neurotrophins, or other morphogens, proper activation of canonical intracellular signaling pathways including ERK and Akt kinase cascades is critical (Zhao *et al*., 2008). Studies showed that compounds which modulate these pathways promote NPC proliferation and self-renewal (Harada *et al*., 2004; Le Belle *et al*., 2011). Recently, some natural products derived from herbal medicines have been reported to promote NPC proliferation (Kim *et al*., 2008; Yabe *et al*., 2010; Lau *et al*., 2012; Lin *et al*., 2012a; Zhuang *et al*., 2012), elucidating the underlying mechanism for the cognition-enhancing effects of these herbal medicines (Lin *et al*., 2012b). Rhizoma *Acori tatarinowii* (AT), the root of *Acori tatarinowii*, has long been a principal medicine in traditional Chinese formulas for the treatment of brain disorders, such as senile dementia, dysmnesia, and stroke. Recent pharmacological studies have revealed that AT possesses neuroprotective effects (Cho *et al*., 2000; Irie & Keung, 2003) and improves learning and memory in aged, dysmnesia murines (Zhang *et al*., 2007; Kim *et al*., 2009) and ischemic rats (Lee *et al*., 2003). However, whether and how AT influences NPC proliferation and self-renewal is not known.

In this study, we reported that AT treatment enhanced hippocampal neurogenesis in wild-type and transgenic AD model mice as well as aged mice. AT and its active constituents, asarones, can promote NPC proliferation both *in vivo* and *in vitro*. Treatment with AT or asarones leads to the activation of ERK cascade rather than Akt cascade. Our results thus suggest that AT and asarones may serve as therapeutic agents to promote neurogenesis against cognitive decline associated with aging and neurodegenerative disorders.

## Results

### AT enhances NPC proliferation in adult mouse brain

**Fig 1:**
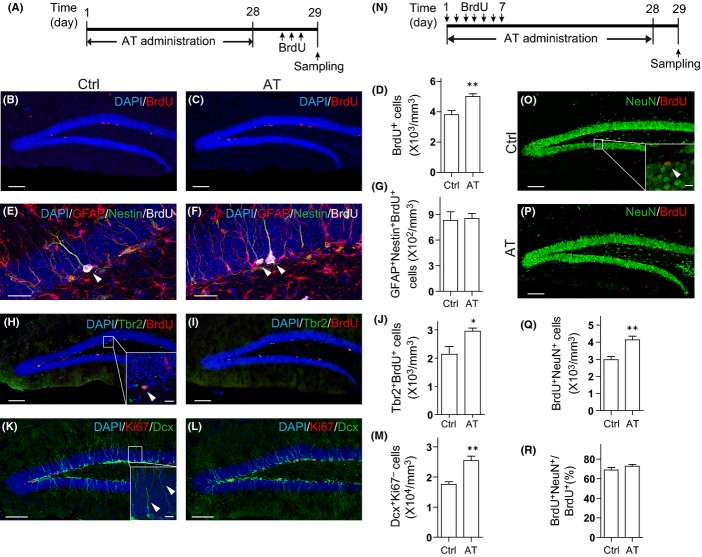
The extract of Rhizoma *Acori tatarinowii* (AT) promotes neural progenitor cell (NPC) proliferation and neurogenesis in adult mouse hippocampus. (A) Diagram depicting the experimental design employed for investigating NPC proliferation. (B, C) Immunostaining for BrdU (red) and DAPI (blue) in coronal hippocampal dentate gyrus (DG) sections from mice treated with (B) vehicle (Ctrl) and (C) AT as indicated in (A). Scale bars, 100 μm. (D) Quantification of BrdU^+^ cells from sections as in (B, C). *N *= 5 to 6 per group. (E, F) Immunostaining for GFAP (red), Nestin (green), and BrdU (grey) in DG sections from mice treated with (E) vehicle (Ctrl) and (F) AT. Scale bars, 25 μm. (G) Quantification of GFAP^+^Nestin^+^BrdU^+^ cells. *N *= 5 to 6 per group. (H, I) Tbr2 (green), BrdU (red), and DAPI (blue) co-staining of DG sections from mice treated with (H) vehicle (Ctrl) and (I) AT. Scale bars, 100 μm. (J) Quantification of Tbr2^+^BrdU^+^ cells. *N *= 5 to 6 per group. (K, L) Ki67 (red), Dcx (green), and DAPI (blue) co-staining of DG sections from mice treated with (K) vehicle (Ctrl) and (L) AT. Scale bars, 100 μm. (M) Quantification of Dcx^+^Ki67^−^ cells. *N *= 5 to 6 per group. (N) Diagram depicting the experimental design employed for examining NPC differentiation. (O, P) BrdU (red) and NeuN (green) staining of DG sections from mice treated with (O) vehicle (Ctrl) and (P) AT. The experiment was performed as depicted in (N). Scale bars, 100 μm. (Q) Quantification of BrdU^+^NeuN^+^ cells from sections as in (O, P). *N *= 7 per group. (R) Proportion of BrdU^+^NeuN^+^ cells in BrdU^+^ cells. *N *= 7 per group. Insets were images of high magnification with scale bars of 10 μm. Quantifications are presented as mean ± SEM; *P < 0.05, **P < 0.01, analyzed by two-tailed *t*-test.

Rhizoma *Acori tatarinowii* has been applied to patients with cognitive deficits caused either by aging or by neurodegenerative diseases. Besides, it has been considered to have general cognition-enhancing effects. Therefore, we first investigated whether AT treatment could be beneficial to neurogenesis in wild-type (WT) mouse hippocampus. We prepared the AT extracts as described elsewhere (Lim *et al*., 2012). The extraction yield is ∼10%. The quality control of each batch was performed with high-performance liquid chromatography (HPLC). Eight-week-old C57BL/6 mice were chronically administrated with 100 μL vehicle (0.8% Tween-80 in water) or extracts that equal to 200 mg Rhizoma *Acori tatarinowii* per 20 g mouse body weight by gavage once per day for 28 days. Mitotic cells were labeled by three bromodeoxyuridine (BrdU) injections on the last day as previously described (Encinas *et al*., 2011) (Fig.1A). Histology analysis showed that a majority of the BrdU-positive cells were localized in the subgranular zone (SGZ) where hippocampal NPCs are localized (Fig.1B). Compared with vehicle treatment, AT treatment increased the number of BrdU-positive proliferating NPCs by 31% (Fig.1B–D). Immunostaining of the proliferation marker Ki67 in the SGZ showed similar results (Figs1K,L, and S1B). We have not observed a significant change in granule cell layer volume following AT administration (Fig. S1A). It is reported that there are two types of adult neural stem cells in SGZ, radial glia-like cells and nonradial NPCs, both of which are capable of proliferation but might possess reciprocal linage relationships (Suh *et al*., 2007). We further assessed the alteration of the radial glia-like stem cells and the nonradial progenitors. We found that Tbr2 and BrdU double-positive proliferating nonradial progenitors were increased by AT treatment (Fig.1H–J), whereas the number of GFAP^+^/Nestin^+^/BrdU^+^ proliferating radial glia-like stem cells were not altered significantly (Fig.1E–G). We did not observe a significant change in Sox2^+^ cell number in SGZ, either, suggesting that AT administration might not affect the NPC pool (Fig. S1C–E). Neuroblasts and immature neurons in the dentate gyrus (DG) were visualized by immunostaining with antibodies against doublecortin (Dcx). The number of Dcx^+^/Ki67^+^ neuroblasts and Dcx^+^/Ki67^−^ immature neurons was both significantly increased by AT treatment (Figs1K–M and S1F–G). The survival of Dcx^+^ cells was not altered following AT treatment, as was revealed by TUNEL assay (Fig. S1H). These results indicate that NPC proliferation was promoted by AT administration.

To further assess the neurons newly generated from NPCs, we labeled proliferating NPCs by daily BrdU injections on the first 7 days of AT administration and sacrificed the mice 28 days after the first BrdU injection (Fig.1N). By examining the expression of the mature neuronal marker NeuN in BrdU-retaining cells, we found that there were more BrdU/NeuN double-positive neurons in the AT-treated mouse brains compared to that in the control mouse brains (Fig.1O–Q). A fraction of these BrdU/NeuN double-positive neurons in AT-treated mice showed expression of c-Fos, indicating the activation of these newborn neurons (Fig. S1I). However, the proportion of NeuN and BrdU double-positive cells in the total BrdU^+^ cells were not altered following AT treatment (Fig.1R), suggesting AT treatment has not affected neuronal lineage commitment. Further, we found that there was no significant alteration of GFAP/BrdU double-positive astrocyte population following AT treatment, which indicates that the gliogenesis has not been affected (Fig. S1J). Together, these results show that AT treatment promotes NPC proliferation and leads to enhanced neurogenesis in the adult mouse hippocampus.

### AT promotes neurogenesis in aged and transgenic AD mouse brain

**Fig 2:**
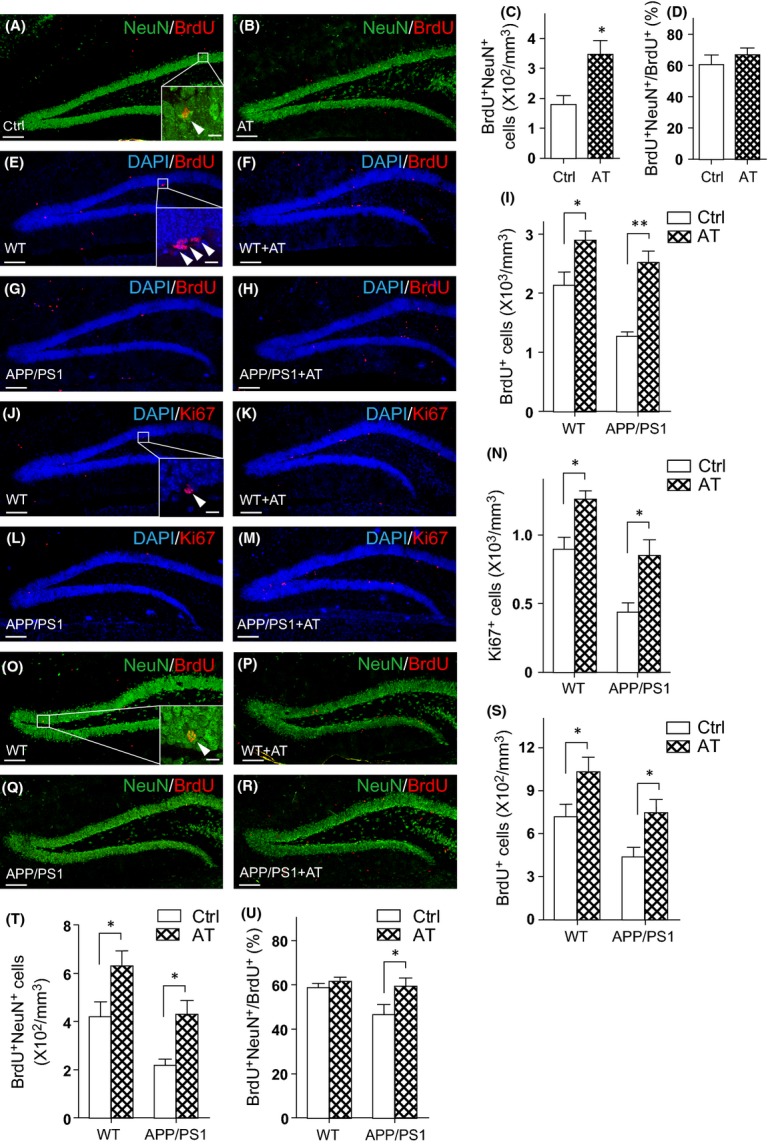
The extract of Rhizoma *Acori tatarinowii* (AT) promotes hippocampal neurogenesis in aged and transgenic AD mice. (A, B) 18- to 23-month-old mice were orally administrated with (A) vehicle (Ctrl) and (B) AT for 28 days and were injected with BrdU on the first 7 consecutive days. The experiment was performed as depicted in (Fig.1N). DG sections were immunostained for BrdU (red) and NeuN (green). (C) Quantification of BrdU^+^NeuN^+^ cells from sections as in (A, B). *N *= 7 per group. (D) Proportion of BrdU^+^NeuN^+^ cells in BrdU^+^ cells. *N *= 7 per group. (E–H) Immunostaining for BrdU (red) and DAPI (blue) in DG sections from wild-type (WT) and APP/PS1 mice treated with vehicle (Ctrl) and AT for 17 days and injected with BrdU on the last 7 days. (I) Quantification of BrdU^+^ cells from sections as in (E–H). *N *= 6 to 8 per group. (J–M) Ki67 staining (red) and DAPI (blue) counterstain of DG sections from wild-type and APP/PS1 mice treated with vehicle (Ctrl) and AT. (N) Quantification of Ki67^+^ cells from sections as in (J–M). *N *= 6 to 8 per group. (O–R) Immunostaining for BrdU (red) and NeuN (green) in the DG sections from wild-type and APP/PS1 mice treated with vehicle (Ctrl) and AT. The experiment was performed as depicted in (Fig.1N). (S) Quantification of BrdU^+^ cells from the sections as in (O–R). *N *= 5 per group. (T) Quantification of BrdU^+^NeuN^+^ cells. *N* = 5 per group. (U) Proportion of BrdU^+^NeuN^+^ cells in BrdU^+^ cells. *N *= 5 per group. Quantifications are presented as mean ± SEM; *P < 0.05, **P < 0.01, analyzed by one-way ANOVA followed by Fisher’s protected least significant difference test; scale bars, 100 μm. Insets were images of high magnification with scale bars of 10 μm.

We then asked whether AT treatment can also promote hippocampal NPC proliferation in aging and neurodegenerating mouse brains. To address these questions, we first treated aged mice (age at 18–23 months) with AT. We injected these aged mice with BrdU once per day for seven consecutive days and treated them with AT or vehicle for 28 days. In these aged mouse brains, there were significantly less BrdU/NeuN double-positive neurons compared to that in the brains of the 8-week-old adult mice (Fig.2A vs. Fig.1O). Interestingly, we found that AT treatment considerably augmented the number of BrdU/NeuN double-positive neurons (∼90%, Fig.2A–C), while the proportion of NeuN/BrdU double-positive cells in the total BrdU^+^ cells was not altered (Fig.2D). These data indicate that AT treatment enhances neurogenesis in aged mice.

Then, 8- to 12-month-old middle-aged APP/PS1 mice and their wild-type (WT) littermates were administrated with AT or vehicle followed by BrdU injections. Compared with that in the brain of 8-week-old mice, there were less BrdU-positive cells in brains of 8- to 12-month-old wild-type mice (Fig.2E vs. Fig.1B). Further, as reported previously (Taniuchi *et al*., 2007), the number of BrdU-positive proliferating cells in APP/PS1 mouse brains were remarkably decreased by 40% (Fig.2E,G,I), indicating deficits in NPC proliferation in these AD transgenic mice. We found that AT treatment exerted a modest increase in NPC proliferation by 36% in wild-type mouse brain (Fig.2E,F,I). Furthermore, in APP/PS1 mouse brain, AT treatment markedly increased NPC proliferation by 98% (Fig.2G,H,I). Immunostaining against Ki67 also showed similar results (Fig.2J–N). The neurogenesis in APP/PS1 mice treated with AT has also been monitored. For this, we injected the APP/PS1 mice and their wild-type littermates with BrdU and treated them with AT for 28 days. We found substantially fewer BrdU-positive cells as well as lower proportion of BrdU/NeuN double-positive neurons in the DG region of APP/PS1 mice compared with that in their wild-type littermates (Fig.2O,Q,S–U). We observed increased BrdU-positive and NeuN/BrdU double-positive cells as well as raised proportion of NeuN/BrdU double-positive cells in the AT-treated APP/PS1 mouse brains (Fig.2O–U), indicating AT treatment increased the newborn cell survival and the number of newly generated neurons. These data indicate that AT treatment retards deficits of NPC proliferation and neurogenesis in neurodegenerating AD model mice.

### AT promotes NPC proliferation *in vitro*

**Fig 3:**
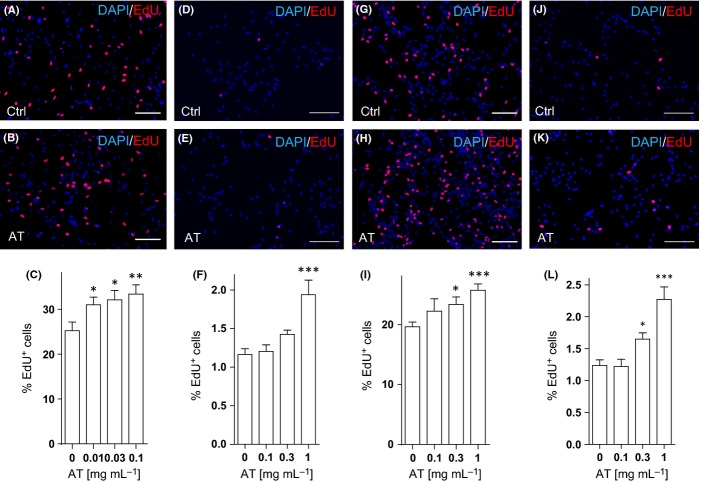
The extract of Rhizoma *Acori tatarinowii* promotes proliferation of neural progenitor cells (NPCs) *in vitro*. (A–C) Monolayer adult hippocampal NPC cultures were treated with AT of different concentrations for 24 h in the reduced growth factor medium (1 ng mL^−1^ EGF and 1 ng mL^−1^ bFGF). EdU was added 2 h prior to fixation. Representative images of EdU (red) and DAPI (blue) stainings in the culture treated with DMSO (Ctrl, A) or 0.1 mg mL^−1^ AT (B) were shown. The percentage of EdU^+^ cells among total cells in the culture (C) was determined. (D–F) Monolayer adult hippocampal NPC cultures were treated with AT of different concentrations for 14 h in the absence of EGF or bFGF. EdU was added 2 h prior to fixation. Representative images of EdU (red) and DAPI (blue) stainings in the culture treated with DMSO (Ctrl, D) or 1 mg mL^−1^ AT (E) were shown. (F) Quantification of EdU^+^ cells (as a percentage of total cells) in the culture. (G–I) Monolayer embryonic neural precursors were treated with AT for 24 h in the reduced growth factor medium. EdU was added 2 h prior to fixation. Representative images of EdU (red) and DAPI (blue) stainings in the culture treated with DMSO (Ctrl, G) or 1 mg mL^−1^ AT (H) were shown, and the percentage of EdU^+^ cells among total cells in the culture (I) was determined. (J–L) Embryonic neural precursors were treated with AT of different concentrations for 14 h in the absence of EGF or bFGF. EdU was added 2 h prior to fixation. Representative images of EdU (red) and DAPI (blue) stainings in the culture treated with DMSO (Ctrl, J) or 1 mg mL^−1^ AT (K) were shown. (L) Quantification of EdU^+^ embryonic neural precursors (as a percentage of total cells) in the culture. Quantifications are presented as mean ± SEM of eight independent experiments; *P < 0.05, **P < 0.01, ***P < 0.001, analyzed by one-way ANOVA followed by Fisher’s protected least significant difference test; scale bars, 100 μm.

To assess the direct effects of AT on NPCs, we derived NPCs from adult mouse hippocampi. About 90% of total cells in the culture were identified as Nestin- and Sox2-positive NPCs (Fig. S2A). The adherent NPC culture rapidly expanded in the medium supplemented with 20 ng mL^−1^ EGF and 10 ng mL^−1^ bFGF. It has been shown that in aging or degenerating brains, decline of the growth factors’ supply along with an increase in quiescence regulators may contribute to decreased neurogenesis (Shetty *et al*., 2005; Tang *et al*., 2009). Therefore, we reduced the supplementary of EGF and bFGF in the NPC culture to mimic such physiological or pathological conditions and examined the effects of AT on NPC proliferation by EdU incorporation. As shown in Fig.3A, about 20% of total cells were EdU-positive in the culture media with 1 ng mL^−1^ EGF and bFGF and this proportion dropped to 1% in the culture media without EGF and bFGF (Fig.3A,D). AT treatment resulted in a dose-dependent increase of EdU incorporation in NPCs under these culture conditions (Fig.3A–F) (∼30% increase under the reduced growth factor condition and ∼65% increase under the no growth factor condition). The proliferation of neural precursors derived from embryonic mouse cortex was also enhanced by AT treatment in a dose-dependent manner (Figs S2B and 3G–L).

On the other hand, consistent with the observation *in vivo* (Fig.1R), the percentage of Tuj1- or GFAP-positive cells cultured under the differentiation condition in the presence of AT was not significantly different from that of the control group (Fig. S2C,D), indicating that AT treatment did not affect NPC lineage commitment *in vitro*.

### Asarones are major active constituents of AT in regulating NPC proliferation

**Fig 4:**
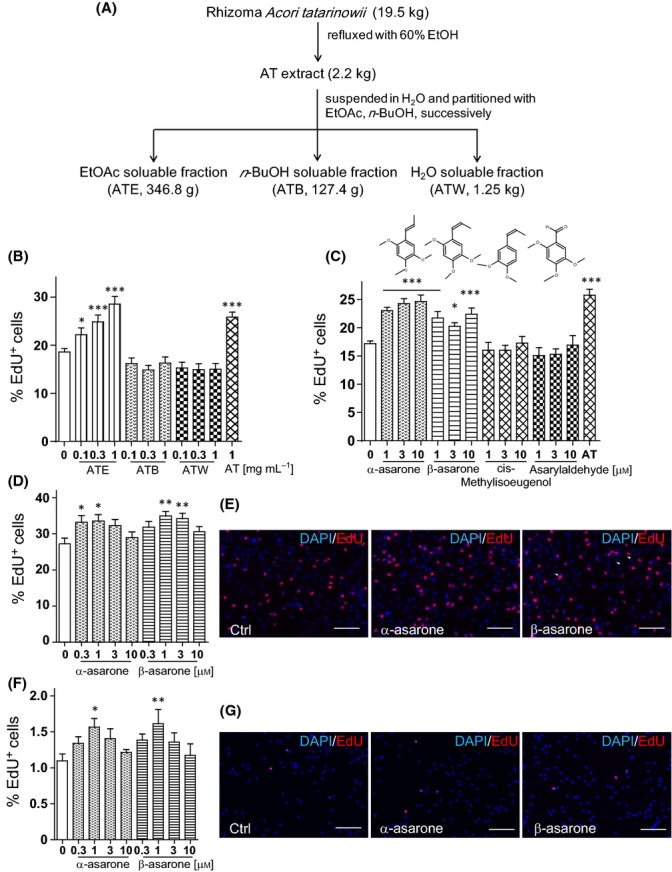
Asarones are active constituents of Rhizoma *Acori tatarinowii* for enhancing neural progenitor cell (NPC) proliferation. (A) Extraction and fractionation scheme of the Rhizoma *Acori Tatarinowii*. (B) Percentage of EdU^+^ cells in the embryonic neural precursor culture treated with the three fractions in the reduced growth factor medium (1 ng mL^−1^ EGF and 1 ng mL^−1^ bFGF). (C) Percentage of EdU^+^ embryonic neural precursors treated with α-asarone, β-asarone, and two analogues in ATE. (D) Percentage of EdU^+^ cells in the adult hippocampal NPC cultures treated with or without asarones. (E) Representative images of EdU^+^ (red) staining and DAPI (blue) counterstain in adult hippocampal NPC cultures treated with DMSO (Ctrl), 1 μm α-asarone, or 1 μm β-asarone. (F) Percentage of EdU^+^ cells in the adult hippocampal NPC culture treated with or without asarones in the absence of growth factors. (G) Representative images of EdU^+^ (red) staining and DAPI (blue) counterstain in adult hippocampal NPC cultures treated with DMSO (Ctrl), 1 μm α-asarone, or 1 μm β-asarone in the absence of growth factors. Quantifications are presented as mean ± SEM of eight independent experiments; *P < 0.05, **P < 0.01, ***P < 0.001, analyzed by one-way ANOVA followed by Fisher’s protected least significant difference test; scale bars, 100 μm.

To identify the active constituents, AT was separated into three fractions (Fig.4A). We examined EdU incorporation of embryonic neural precursors under the reduced growth factor condition and the no growth factor condition (Figs4B and S4A). The concentrations of each fraction were calculated according to the mass yield and were presented as weight/volume (w/v) of original raw material. As shown in Figs4B and S4A, the treatment of the EtOAc soluble fraction (ATE) increased the percentage of EdU^+^ cells in a dose-dependent manner similar to that of AT treatment. The ATB or ATW fractions, that is, n-BuOH and H_2_O soluble fractions, did not significantly affect EdU incorporation compared with the control culture. Thus, we identified ATE as the major active fraction of AT for promoting NPC proliferation.

To further determine the active constituents in ATE, HPLC analysis was performed (Fig. S3A–D). There were two major chemical constituents enriched in the ATE fraction (∼20% w/w), β-asarone and α-asarone (Fig. S3B). In the crude AT extracts, there are ∼3% (w/w) of asarones (Fig. S3A). In ATB and ATW fractions, only a trace amount of β-asarone or α-asarone was detected (Fig. S3C,D).

Then, we assessed the effects of asarones on NPC proliferation. As shown in Figs4C and S4B, α-asarone and β-asarone treatment increased EdU incorporation in a dose-dependent manner. In contrast, the treatment with cis-methylisoeugenol or asarylaldehyde, two analogues of asarones identified in ATE, showed no significant enhancement for NPC proliferation. Further, the treatment with α-asarone and β-asarone also promoted the proliferation of adult hippocampal NPCs in a dose-dependent manner (Fig.4D–G). To test whether asarones increase the proliferative capacity of different cell types, especially that of cancer cells, we cultured SH-SY5Y, a neuroblastoma cell line with or without asarones under the serum-free culture condition. Results of MTT analysis showed that different from the treatment with EGF or bFGF, asarones did not significantly affect SH-SY5Y proliferation (Fig. S5), suggesting that asarones might selectively promote NPC proliferation. Further, similar to AT treatment, asarone treatment did not alter the differentiation potency of NPCs as the proportions of Tuj1^+^ cells and GFAP^+^ cells differentiated from asarone-treated NPCs were comparable to those from control NPCs (Fig. S4C). In primary neurons from postnatal mouse cortex and hippocampus, a dose-dependent increase in neurite length of the AT and asarone treatment was observed, whereas the neurite number did not alter (Fig. S4D–F). Interestingly, the maximum effects of asarones on the enhancement of neurite outgrowth occurs at a concentration of 0.1–0.3 μm, which is lower than that for promoting adult hippocampal NPC proliferation (0.3–3 μm), implying different mechanisms may be involved. These results indicate α-asarone and β-asarone are the major active constituents in AT for promoting NPC proliferation *in vitro*.

### Asarones promote hippocampal NPC proliferation and neurogenesis *in vivo*

**Fig 5:**
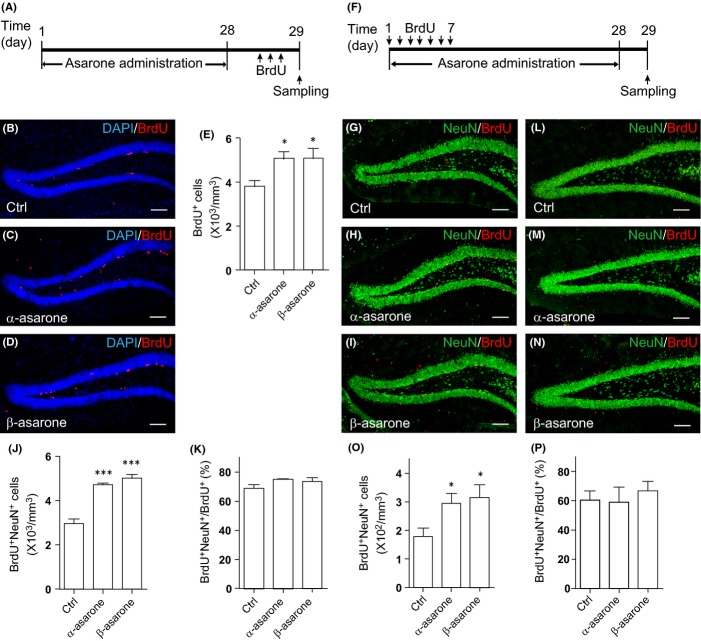
Asarones enhance neural progenitor cell (NPC) proliferation and neurogenesis in mouse hippocampus. (A) Diagram depicting the experimental design employed for examining NPC proliferation. (B–D) BrdU (red) staining and DAPI (blue) counterstain of DG sections from 8-week-old mice treated with (B) vehicle (Ctrl), (C) α-asarone, or (D) β-asarone. The experiment was performed as depicted in (A). (E) Quantification of BrdU^+^ cells as in (B–D). *N* = 5–6 per group. (F) Diagram depicting the experimental design employed for examining NPC differentiation. (G–I) BrdU (red) and NeuN (green) stainings of DG sections from mice treated with (G) vehicle (Ctrl), (H) α-asarone, or (I) β-asarone. The experiment was performed as depicted in (F). (J) Quantification of BrdU^+^NeuN^+^ cells in (G–I). *N *= 4–7 per group. (K) Proportion of BrdU^+^NeuN^+^ cells in BrdU^+^ cells. *N *= 4–7 per group. (L–N) BrdU (red) and NeuN (green) stainings of DG sections from 18- to 23-month-old mice treated with (L) vehicle (Ctrl), (M) α-asarone, or (N) β-asarone. The experiment was performed as depicted in (F). (O) Quantification of BrdU^+^NeuN^+^ cells in (L–N). *N *= 5–7 per group. (P) Proportion of BrdU^+^NeuN^+^ cells in BrdU^+^ cells. *N *= 5–7 per group. Quantifications are presented as mean ± SEM; *P < 0.05, ***P < 0.001, analyzed by one-way ANOVA followed by Fisher’s protected least significant difference test; scale bars, 100 μm.

To test whether asarones promote adult hippocampal neurogenesis *in vivo* similar to AT, 8-week-old C57BL/6 mice were administrated with α-asarone or β-asarone orally for 28 days and proliferative cells were labeled by BrdU injections (Fig.5A). Compared with that in vehicle-treated mice, there were an increased number of BrdU-positive proliferating NPCs in the SGZ of α-asarone- and β-asarone-treated mice (Fig.5B–E). Immunostaining against Ki67 showed similar results (Fig. S6I–L). Similar to AT treatment, asarone treatment preferentially enhanced the proliferation of the Tbr2-positive nonradial progenitors (Fig. S6E–H), while that of the GFAP/ Nestin double-positive radial glia-like stem cells has not been altered (Fig. S6A–D). The NPC pool represented by Sox2-positive cells in SGZ was not affected, either (Fig. S6O). Asarone treatment increased the number of Dcx^+^/Ki67^+^ neuroblasts and Dcx^+^/Ki67^−^ immature neurons (Fig. S6I–K,M,N), whereas the survival of these cell populations has not been affected (Fig. S6P). To further assess the neurons newly generated from NPCs, we applied BrdU injections on the first 7 days of asarone administration and sacrificed the mice 28 days later (Fig.5F). Histology revealed that asarone treatment increased the number of BrdU/NeuN double-positive neurons in the mouse brain compared with that in vehicle-treated mice (Fig.5G–J). A small proportion of the BrdU/NeuN double-positive neurons showed expression of c-Fos, indicating a functional activation (Fig. S6Q). However, the proportion of NeuN^+^ cells in BrdU-retaining cells did not change following asarone treatment (Fig.5K), suggesting that similar to AT, the treatment with asarones has not affected neuronal lineage commitment. Interestingly, we found that GFAP/BrdU double-positive cells were also increased following asarone treatment, indicating asarones also promoted gliogenesis (Fig. S6R). Together, these results show that asarone and AT treatment promote NPC proliferation and lead to enhanced neurogenesis in adult mouse hippocampus.

Neurogenesis in asarone-treated aged mice was further assessed. For this, we labeled proliferating cells in the 18- to 23-month-old mice by BrdU injections for seven consecutive days and treated the mice with vehicle or asarones for 28 days. Similar to AT treatment, asarone treatment leads to an increase in BrdU/NeuN double-positive neurons by ∼70% (Fig.5L–O), while neuronal lineage commitment did not alter (Fig.5P). These data indicate that asarone treatment enhances neurogenesis in aged mice.

Both cognition and neurogenesis decline with aging. To investigate whether AT and asarone treatment was associated with a functional behavioral consequence, we applied a novel object recognition test to assess hippocampal-dependent recognition memory in the aged mice. During training phase, vehicle-, AT-, and asarone-treated mice showed an equal preference index for the two identical objects (Fig. S6S). Twenty-four hours later, the vehicle-treated mice were unable to discriminate the novel object from the familiar one, as they fail to explore the novel objects above the 50% chance level (Fig. S6T). In contrast, the AT- and asarone-treated mice showed an elevated preference to the novel object over the familiar one, indicating they had substantial retention memory (Fig. S6T). These results indicate that AT and asarones reduced the deficits in recognition memory of aged mice. It is of note that the correlation between neurogenesis and cognitive performance in old age is still obfuscated (Bizon & Gallagher, 2003; Drapeau *et al*., 2003; Merrill *et al*., 2003; Seib *et al*., 2013). Our results showed AT and asarone treatment in the aged mice leads to both enhanced neurogenesis and improved recognition memory, which might therefore provide a unique system to further elucidate the molecular and cellular connection between neurogenesis and cognitive performance.

### AT and asarones activate ERK cascade but not Akt cascade

**Fig 6:**
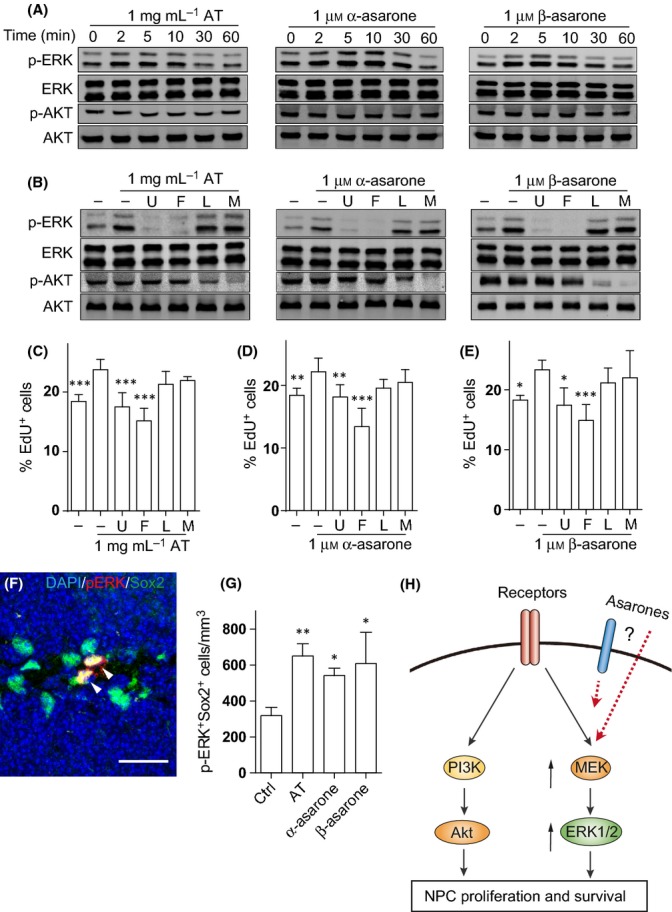
The extract of Rhizoma *Acori tatarinowii* (AT) and asarones activate ERK cascade but not Akt cascade to promote neural progenitor cell (NPC) proliferation. (A) Embryonic neural precursors were cultured in reduced growth factor medium (1 ng mL^−1^ EGF and 1 ng mL^−1^ bFGF) for 24 h and were treated with 1 mg mL^−1^ AT, 1 μm α-asarone, or 1 μm β-asarone for the indicated time. Protein samples were analyzed on Western blots using antibodies against the phosphorylated ERK1/2 and Akt. Blots were probed for total ERK and Akt as loading controls. Four independent experiments showed similar results. (B) MEK inhibitor U0126 (U; 0.1 μm), ERK inhibitor FR180204 (F; 10 μm), PI3K inhibitor LY294002 (L; 10 μm), or Akt inhibitor MK-2206 (M; 10 μm) was added to the embryonic neural precursor culture. After 30 min, cells were treated with AT or asarones for 5 min. Blots of the phosphorylated ERK1/2, total ERK, phosphorylated Akt, and Akt were shown. Four independent experiments showed similar results. (C–E) Embryonic neural precursors cultured in reduced growth factor medium were pretreated with MEK inhibitor U0126 (U; 0.1 μm), ERK inhibitor FR180204 (F; 10 μm), PI3K inhibitor LY294002 (L; 10 μm), or Akt inhibitor MK-2206 (M; 10 μm) for 30 min. (C) AT, (D) α-asarone or (E) β-asarone was then added, and the cells were incubated for additional 24 h. EdU incorporation on the last 2 h was visualized with staining and quantified. All the groups in (C–E) were compared with the cell culture treated with (C) AT, (D) α-asarone, or (E) β-asarone alone, respectively. *N *= 5 independent experiments. (F–G) Mice were treated with vehicle (Ctrl), AT, or asarones for 5 days. DG sections were immunostained for phosphorylated ERK1/2 (F, red) and Sox2 (F, green), and the p-ERK^+^Sox2^+^ cells were quantified as in (G). *N *= 4–6 per group. Scale bars, 20 μm. (H) Schematic diagram shows that asarones promote NPC proliferation through the activation of ERK cascades. Quantifications are presented as mean ± SEM; *P < 0.05, **P < 0.01, ***P < 0.001; analyzed by one-way ANOVA followed by Fisher’s protected least significant difference test.

Studies show that proper activation of ERK and/or Akt kinase cascades is critical for NPC proliferation and self-renewal. Thus, we monitored the activity of these kinase cascades on immunoblots. As shown in Fig.6A, following AT and asarone treatment, cellular ERK phosphorylation increased, peaking at 2–10 min, and slowly declined to the basal level in 30 min. However, we did not observed any significant alteration on the Akt phosphorylation levels (Fig.6A). Further, the activation of ERK by either AT or asarones could be blocked by the MEK inhibitor U0126 and the ERK inhibitor FR180204 but not the PI3K inhibitor LY294002 or the Akt inhibitor MK-2206 (Fig.6B). To further test whether promotion of NPC proliferation by AT or asarones depended on the activation of ERK cascade, NPCs were treated with AT or asarones in the presence of U0126, FR180204, LY294002, or MK-2206. As shown in Fig.6C–E, the treatment with U0126 and FR180204, but not that with LY294002 and MK-2206, largely blocked AT- or asarone-promoted NPC proliferation, suggesting MEK/ERK cascade rather than PI3K/Akt cascade is required for promoting NPC proliferation by AT and asarone treatment. To confirm the role of ERK signaling in asarones promoting NPC proliferation *in vivo*, we treated 11-week-old C57BL/6 mice with AT or asarones for 5 days. The number of BrdU-positive proliferating cells in the SGZ was increased by 25% compared to that of the control mice, indicating that AT and asarone treatment gave rise to a direct and fast promotion of NPC proliferation (Fig. S7A–E). Then, we co-stained phosphorylated ERK and Sox2 in the DG of AT- and asarone-treated mice (Fig.6F). We found a significant increase of pERK-positive NPCs in AT- and asarone-treated mice along with the enhanced NPC proliferation, indicating enhanced ERK activation in the NPCs of the mice treated with AT and asarones (Fig.6G).

## Discussion

Neurogenesis declines during aging and neurodegenerative disease process (Zhao *et al*., 2008). Transplantation of exogenous NPCs and mobilization of endogenous NPCs have been considered as promising cell therapeutic strategies against neurodegeneration. However, only a limited number of chemicals have been found to promote neurogenesis in spite of the huge efforts spent through high-throughput and high-content screening so far (Li *et al*., 2012). Here, we took the advantage of herbal medicines with clinically proved effects. We chose AT, a Chinese herbal medicine with known cognitive enhancing effects, and applied systematic approaches to test its roles in neurogenesis. Further, we adopted stepwise fractionation of AT and identified the active chemical constituents, asarones, for promoting neurogenesis *in vitro* and *in vivo*.

In addition to our findings, β-asarone has been reported to protect the progeny of NPCs from neurotoxicity induced by β-amyloid (Geng *et al*., 2010; Li *et al*., 2010). A recent report demonstrated inhibitory effects of α-asarone on pro-inflammatory cytokine production and microglia activation in systemic lipopolysaccharide-treated mice (Shin *et al*., 2014). These reports together with our results suggest a multitarget function of AT and asarones, which might have desirable advantages against multifactorial neurodegenerative process and diseases including AD.

MEK/ERK and PI3K/Akt are two cascades critical for regulating NPC functions. Activation of MEK/ERK cascade is primarily responsible for cell proliferation, while the PI3K/Akt cascade is important not only for proliferation, but also for the differentiation and survival of NPCs (Otaegi *et al*., 2006; Kalluri *et al*., 2007; Goetz & Mohammadi, 2013). Our results indicated that AT and asarones function through ERK but not through Akt cascades. Consistently, promotion of NPC proliferation by AT and asarone treatment can be blocked by the inhibition of either MEK or ERK activation. On the other hand, the differentiation potential of NPC was not altered by either AT or asarone treatment. Although the exact molecular targets of asarones in promoting NPC proliferation and self-renewal are still unclear, our study provided preliminary hints for their underlying mechanisms (Fig.6H). On one hand, considering that asarones promote proliferation of NPCs, it is possible that they could modulate the activation of membrane proteins such as receptors. On the other hand, asarones may cross membranes and directly act on their intracellular targets for the activation of MEK/ERK cascade, which remains to be investigated. Moreover, asarones did not promote the proliferation of neuroblastoma cell line SH-SY5Y and even inhibit the proliferation of some cancer cells (Zou *et al*., 2012; Liu *et al*., 2013), suggesting the specificity of asarone functions.

Neurogenesis decreases markedly with aging. However, under the same *in vitro* culture conditions, NPCs from adult and aged brains show similar proliferation potency, indicating that the environment in the aged brain, rather than the intrinsic NPC properties, is most likely to be responsible for declined neurogenesis (Ahlenius *et al*. 2009). Studies show that in aging and neurodegenerating brains, the growth factor supply dramatically declines, which contributes to the decreased neurogenesis and related cognitive impairment (Shetty *et al*., 2005; Tang *et al*., 2009). Interestingly, we found AT and asarone treatment enhanced the NPC proliferation *in vitro* under culture conditions with insufficient growth factor supply. We did not observe this promoting effect of AT or asarones on NPCs that cultured in the medium supplemented with growth factors as suggested in standard NPC culture protocols (Brewer & Torricelli, 2007), suggesting that the beneficial effects of AT and asarones may be related to the physiological and pathological conditions in which growth factor supply was deficient. Consistently, we also observed an even more significant increase in NPC proliferation by AT in the aged and AD mice than in the middle-aged and young mice. Thus, our results not only provide AT and its active constituent asarones as potential NPC proliferation promoting agents, but further suggest that a modified culture strategy with less growth factor supplements that mimic pathological conditions may be valuable in assessing the regenerative effects of drugs against neurodegeneration.

## Experimental procedures

### Animals

C57BL/6 mice were obtained from Shanghai Laboratory Animal Center (Chinese Academy of Sciences). APP/PS1 transgenic mice (Jackson Laboratory, stock number 004462) express a chimeric mouse/human amyloid precursor protein containing the K595N/M596L Swedish mutations (APPswe) and a human presenilin 1 with a deletion of exon 9. Heterozygous mice were maintained by crossing with C57BL/6 mice. The experiment procedures for the use and care of the animals were approved by the Ethics Committees of the Shanghai Institutes for biological Sciences, Chinese Academy of Sciences. All mice were given *ad libitum* access to food and water.

### Preparation and fractionation of extracts from Rhizome *Acori Tatarinowii*

The dried rhizomes (19.5 kg) of *Acori tatarinowii* L. from Hunan Province in China (purchased from PuraPharm Corporation, Guangxi, China) were refluxed three times with 70 L 60% EtOH for 2 h each time as described elsewhere (Lim *et al*., 2012). The extracts were combined and concentrated by evaporation. About 2.2 kg of final crude extracts were obtained and named AT. The extraction efficiency was 11.3% (w/w). AT was then dissolved in 10 L H_2_O. Stepwise fractionation was performed using EtOAc (30 L) and n-butanol (30 L). Organic solvents were removed by vacuum evaporation. The yield of EtOAc soluble fraction (ATE), n-butanol soluble fraction (ATB), and H_2_O soluble fraction (ATW) were 346.8 g (1.8%), 127.4 g (0.7%), and 1.25 kg (6.4%), respectively.

The chromatographic separation and detection of samples was performed on a Waters 2695 system equipped with a UV detector with a Cosmosil column (C18, 5 μm, 250 × 4.6 mm). Column temperature was maintained at 35 °C, and liquid flow rate was set at 1.0 mL min^−1^ with MeOH (Phase A) and H_2_O (Phase B) as mobile phase with a linear gradient: 0–10 min (10–55%, A), 10–20 min (55–60%, A), 20–40 min (60–75%, A), 40–50 min (75–100%, A), and 50–65 min (100%, A).

### Drug administration and BrdU injections

α-asarone, β-asarone (Sigma Aldrich, St Louis, MO, USA), and AT were dissolved in vehicle (H_2_O with 0.8% Tween-80). BrdU (Sigma Aldrich) intraperitoneal injections were applied as described (Encinas *et al*., 2011) and were illustrated in Figs1A,K and 5A,F. For NPC differentiation evaluation, mice were chronically administrated with 100 μL AT extracts that equal to 200 mg Rhizoma *Acori tatarinowii* per 20 g mouse body weight, α-asarone 10 mg kg^−1^, β-asarone 30 mg kg^−1^, or vehicle by gavage once per day for 28 days. The mice were injected with BrdU (50 mg kg^−1^) once per day at the first 7 days of drug administration. 28 days after the first BrdU injection, the animals were transcardially perfused with cold PBS and 4% paraformaldehyde (PFA), successively.

For monitoring NPC proliferation, mice were chronically treated with AT, α-asarone, β-asarone, or vehicle once per day for 28 days. About 150 mg kg^−1^ BrdU were injected three times separated by 3-h intervals on the following day of the last drug administration. The animals were perfused 2 h after the last BrdU injection. To assess the effects of AT on NPC proliferation in AD mice, 8-month-old APP/PS1 mice and their wild-type littermates were treated with vehicle or AT by intragastric gavage once daily for 17 days. BrdU (50 mg kg^−1^) was injected to the mice once per day from day 10 to day 17 of drug administration. The mice were sacrificed 24 h after the last BrdU injection.

### Cell culture

Adult hippocampal NPCs were derived from hippocampi of 6- to 8-week-old C57BL/6 mice and were cultured as neurospheres in NeuroCult NSC basal medium with pro liferation supplement (StemCell Technologies, Inc., Vancouver, BC, Canada) containing 20 ng mL^−1^ EGF and 10 ng mL^−1^ bFGF (Gibco, Frederick, MD, USA) as previously described (Brewer & Torricelli, 2007). Neural progenitor cells were enriched and expanded via serial passages and were used for experiments at passage 6–10.

Embryonic cortical precursors were isolated from cortices of E12.5 C57BL/6 mouse embryos and cultured as neurospheres in DMEM/F-12 (1:1) medium supplemented with B27 supplement (Gibco, Grand Island, NY, USA), 20 ng mL^−1^ EGF, and 10 ng mL^−1^ bFGF as described in Ebert *et al*. (2008). The 6th to 10th passages were used for experiments.

### *In vitro* EdU incorporation assay

For experiments with cells cultured under the conditions with reduced growth factors, neurospheres were dissociated with Accutase (Sigma Aldrich) and cells were seeded at a density of 4 × 10^4^ cells cm^−2^ onto poly-D-lysine (PDL) and laminin-coated 96-well plates in B27-supplemented DMEM/F12 medium (for embryonic neural precursors) or NeuroCult NSC proliferation medium (for adult hippocampal NPCs) containing 1 ng mL^−1^ EGF and 1 ng mL^−1^ bFGF. The next day, the cells were pretreated with or without U0126 (0.1 μm; Sigma Aldrich), LY294002 (10 μm; Sigma Aldrich), MK-2206 (10 μm; Selleck Chemicals, Houston, TX, USA), and FR180204 (10 μm; National Compound Resource Center, Shanghai, China) for 30 min before AT, α-asarone or β-asarone was added. Cells were incubated for 24 h, and EdU (10 μm; Sigma Aldrich) was added for the last 2 h prior to fixation.

For experiments with cells cultured under the conditions without growth factors, neurospheres were dissociated and cells were seeded at a density of 5 × 10^4^ cells cm^−2^ onto PDL and laminin-coated 96-well plates in the culture medium containing 20 ng mL^−1^ EGF and 10 ng mL^−1^ bFGF. The next day, EGF and bFGF were removed and AT, α-asarone, or β-asarone was added. Cells were incubated for additional 14 h, and EdU (10 μm; Sigma Aldrich) was added for the last 2 h prior to fixation.

### *In vitro* NPC differentiation

Neurospheres were dissociated with Accutase, and cells were seeded at a density of 2.5 × 10^4^ cells cm^−2^ onto PDL/laminin-coated 96-well plates in NeuroCult NSC proliferation medium containing 20 ng mL^−1^ bFGF. On the next day, the medium was replaced with NeuroCult NSC basal medium supplemented with differentiation supplement (StemCell Technologies) containing AT or asarones. The differentiation medium was changed every 2 days. 5 days later, the expression of the neuron marker and the astrocyte marker was detected.

### Western blotting

Neural progenitor cells were seeded at 1.2 × 10^5^ cells per well in a 12-well culture plate in B27-supplemented DMEM/F12 containing 1 ng mL^−1^ EGF and 1 ng mL^−1^ bFGF. On the next day, cells were pretreated with U0126 (0.1 μm), LY294002 (10 μm), MK-2206 (10 μm), or FR180204 (10 μm) for 30 min before AT or asarones were added. After treatment for indicated time, cells were washed with cold PBS and lysed with Laemmli’s sample buffer. Cell lysates were separated by SDS/PAGE and transferred onto nitrocellulose membrane. Proteins were labeled with primary antibodies as follows: rabbit monoclonal antibodies against phospho-ERK, ERK, phospho-Akt, or Akt (Cell Signaling Technology, Danvers, MA, USA). The membrane was detected by secondary antibody conjugated to a CW800 fluorescent probe (Rockland Immunochemicals, Gilbertsville, PA, USA) using an infrared imaging system (Odyssey; LI-COR Biosciences, Lincoln, NE, USA).

### Immunostaining

For brain tissue immunostaining, cardiac PBS-perfused mouse brains were further perfused with 4% PFA. The brain tissues collected were postfixed for 24 h and allowed to settle in a 30% sucrose solution for 72 h. Brains were cryosectioned at 30 μm. Eight to nine evenly distributed sections (240 μm apart) including the dentate gyrus (DG) area were selected and were incubated in blocking buffer (10% donkey serum and 0.3% Triton X-100 in PBS) for 45 min at room temperature (RT). Afterward, samples were incubated with primary antibodies at 4 °C overnight and then with appropriate fluorescent probe-conjugated secondary antibodies for 1 h at RT. Nuclei were counterstained with DAPI. The entire dentate gyri were scanned using fluorescence microscope (Olympus BX51; Olympus, Tokyo, Japan), Olympus FV100i (Olympus), or Leica SP-8 (Leica, Mannhein, Germany) confocal microscope. The number of single- or double-stained cells was counted using the Image Pro-Plus software (Media Cybernetics, Silver Spring, MD, USA). For Nestin, Tbr2, Ki67, Sox2, c-Fos, and pERK stainings, antigens were retrieved with citrate buffer (10 mm, pH 6.5) for 20 min at 95 °C before blocking. For BrdU staining, sections were treated in 2 m HCl at 37 °C for 30 min and rinsed in 0.1 m borate buffer (pH 8.5) before blocking. Specific primary antibodies used include rat anti-BrdU (1:2000; AbD Serotec, Oxford, UK), mouse anti-Nestin (1:100; Millipore, Temecula, CA, USA), rabbit anti-GFAP (1:1000; DAKO, Glostrup, Denmark), rabbit anti-Tbr2 (1:200; Abcam, Cambridge, MA, USA), rabbit anti-Ki67 (1:1000; Abcam), goat anti-Dcx (1:200; Santa Cruz Biotechnology, Santa Cruz, CA, USA), goat anti-Sox2 (1:60; R&D systems, Minneapolis, MN, USA), mouse anti-NeuN (1:200; Millipore, Billerica, MA, USA), rabbit anti-c-Fos (1:50; Santa Cruz Biotechnology), and rabbit anti-p-ERK (1:200; Cell Signaling Technology) antibodies. TUNEL staining with *in situ* cell death detection kit was performed following the manufacturer’s protocol (Roche, Mannheim, Germany).

Cells were fixed in 4% PFA for 15 min and permeabilized with 0.1% Triton X-100 in PBS for further immunofluorescent staining. EdU staining was performed following the manufacturer’s protocol (Click-iT; Invitrogen/Molecular Probes, Eugene, OR, USA). Specific primary antibodies used include mouse anti-Nestin (1:1000; Millipore), mouse anti-Tuj1 (1:1000; Convance, Dedham, MA, USA), rabbit anti-GFAP (1:1000; DAKO), and anti-MAP2 (1:500; Millipore). Stained cells were scanned and counted using Operetta high content analysis system (PerkinElmer, Waltham, MA, USA) or Cellomics ArrayScan VTI 700 (Thermo Scientific, Pittsburgh, PA, USA).

### Statistical analysis

All quantified data are presented as mean ± SEM. Results were analyzed by two-tailed *t-*test to determine statistical significance of treatment sets. For multiple comparisons, results were analyzed by one-way analysis of variance (ANOVA) when appropriate with Graphpad Prism 6 software (Graphpad Software, La Jolla, CA, USA). *P*-values less than 0.05 (*P *< 0.05) are considered indicative of significance.

## Supporting Information

**Data S1.** Experimental procedures. **Fig. S1.** Effects of AT on adult hippocampal neurogenesis. **Fig. S2.** Effects of AT and asarones on NPC differentiation *in vitro*. **Fig. S3.** HPLC analysis of AT, ATE, ATB and ATW. **Fig. S4.** Effects of asarones on NPC differentiation and neurite outgrowth. **Fig. S5.** Effects of AT and asarones on neuroblastoma cell line proliferation. **Fig. S6.** Effects of asarones on adult hippocampal neurogenesis. **Fig. S7.** Short-term treatment with AT and asarones promotes hippocampal NPC proliferation.
