# Supplementary material for: A herbal medicine for Alzheimer’s disease and its active constituents promote neural progenitor proliferation
Source: Aging Cell. 2015 May 25;14(5):784–96. doi: 10.1111/acel.12356 (PMC4568966; doi:10.1111/acel.12356)
Supplement: Supplementary file 1 [file acel0014-0784-sd1.pdf]

Supplementary Experimental Procedures

Neurite outgrowth

Primary neurons were isolated from the cortices and hippocampi of newborn C57BL/6 mice and were cultured as described previously (Ahlemeyer & Baumgart-Vogt 2005) . Cells were plated onto poly-D-lysine-coated 96-well plates at the density of 2.5×10^4 cells/mm^2 in DMEM/F12 containing 10% FBS (Invitrogen) and were maintained at 37℃ for four hours. The culture medium was then switched to B27/Neurobasal medium (Invitrogen). The cell cultures were treated with vehicle, AT or asarones from day 3 to day 7 followed by immunostaining for MAP2. Typically, pictures of 100-200 neurons from three separate wells from each experiment were taken and anylzed by Cellomics ArrayScan VTI HSC Reader. Cells with strong MAP2 immunoreactivity labeling neurite (axonal and dendritic) processes were analyzed. Neurites that had lengths above 30 μm were measured.

MTT cell viability assay

SH-SY5Y cells were seeded and cultured in DMEM containing 10% FBS in 96-well plates for 24 h. The culture medium was then replaced by FBS-free DMEM in the presence of vehicle (Ctrl), 1 mg/ml AT, 1 mg/ml ATE, 1 μM α-asarone, 1 μM β-asarone, 20 ng/ml EGF or 10 ng/ml bFGF. The cells were incubated for 2 days and cell viability was then determined by the MTT assay.

Novel object recognition

Tests were performed as previously described (Bevins & Besheer 2006) with modifications. Two identical objects were placed in a box (25 cm×25 cm×25 cm) at the time of the training phase. The mouse was free to explore for 10 min and the time spent exploring each objects was recorded. One day later during the test phase, the mouse was returned to the same box with one familiar object switched to a novel one, and again the time spent exploring each objects was recorded when the animal was free to explore for 10 min. Object exploration time was the length of time when a mouse was sniffing, directing its nose to and pawing the object. The exploration time was recorded in a double-blinded manner. The location preference in the training phase and recognition index in the testing phase were calculated as following: Preference score means the time exploring one object relative to the time exploring the two objects, and Recognition index means the time exploring the novel object relative to the time exploring the two objects.

Short-term drug administration

11-week-old mice were orally administrated with vehicle, AT or asarones once per day for 5 days. 2 hrs after the drug administration on day 5, the mice were injected with BrdU for three times separated by 3 hr intervals. For the purpose of detecting ERK activation in the DG, 1.5 hrs after the last BrdU injection, the mice were treated with the drugs again and were perfused 0.5 hr later.

Supplementary References

Ahlemeyer B , Baumgart-Vogt E (2005). Optimized protocols for the simultaneous preparation of primary neuronal cultures of the neocortex, hippocampus and cerebellum from individual newborn (P0.5) C57Bl/6J mice. *J Neurosci Methods*. **149**, 110-120.

Bevins RA , Besheer J (2006). Object recognition in rats and mice: a one-trial non-matching-to-sample learning task to study 'recognition memory'. *Nat Protoc*. **1**, 1306-1311.

Supplementary Figures

Fig. S1 Effects of AT on adult hippocampal neurogenesis. (A) Measurement of the volume of the granule cell layer (GCL) from the mice treated with vehicle (Ctrl) or AT for 28 days. Coronal hippocampal sections (30 μm thickness) were sliced at 240 μm interval along the anterior-posterior axis and GCL area was outlined on the sections according to DAPI nuclear staining to estimate GCL volume. N = 5 to 6 per group.(B) Quantification of $Ki67^+$ cells from sections as in Fig. 1K, L. N = 5-6 per group. (C-E) Immunohistochemical detection (D-E) and quantification (C) of $Sox2^+$ cells (red) in the DG sections from mice treated with (D) vehicle (Ctrl) or (E) AT as indicated in Fig. 1A. Scale bars, 100 μm. N = 3 per group. (F) Immunostaining for Ki67 (red) and Dcx (green) in the DG as in Fig. 1K. Scale bars, 10 μm. (G) Quantification of Dcx^+Ki67^+ cells from sections as in Fig. 1K. N = 5 to 6 per group. (H) Quantification of Dcx and TUNEL double-stained cells in the DG sections from mice treated with vehicle (Ctrl) or AT. N = 3 per group. (I) Confocal images for c-Fos (blue), BrdU (red) and NeuN (green) co-staining in the DG from the mice treated with AT. The experiment was performed as depicted in Fig. 1N. Scale bars, 10 μm. (J) The experiment was performed as depicted in Fig. 1N. The DG sections from the mice treated with vehicle and AT were immunostained for GFAP, NeuN and BrdU and were quantified for $GFAP^+BrdU^+$ cells. N = 4 to 5 per group. Quantifications are presented as mean ± SEM; *$P < 0.05$, **$P < 0.01$, analyzed by two-tailed t test.

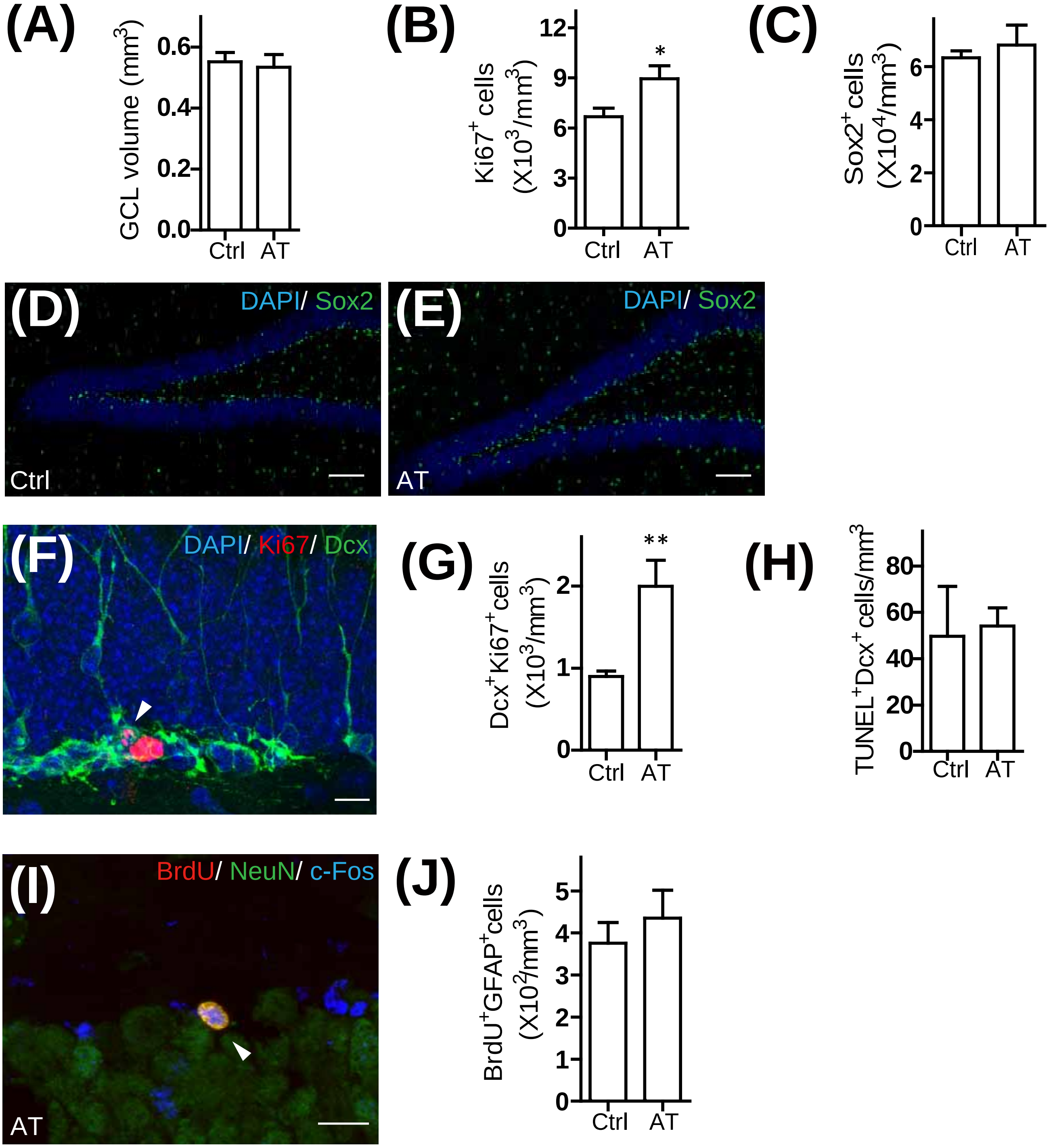

Fig. S1 Mao. et al.

Fig. S2 Effects of AT on NPC differentiation *in vitro*. (A) A representative image of Nestin (green) and Sox2 (red) expression in the adult hippocampal NPC culture. (B) A representative image of Nestin (green) and Sox2 (red) expression in the embryonic neural precursor culture. (C) A representative image of Tuj1 (green, a marker of neurons) and GFAP (red, a marker of astrocytes) expression in adult hippocampal NPC cultures after 5 days under the differentiation conditions. (D) Percentage of Tuj1$^+$ (left) or GFAP$^+$ (right) cells in the adult hippocampal NPC cultures treated with AT of different concentrations after 5 days under the differentiation condition. Quantifications are presented as mean ± SEM of five independent experiments and are analyzed by one-way ANOVA; scale bars, 100 μm.

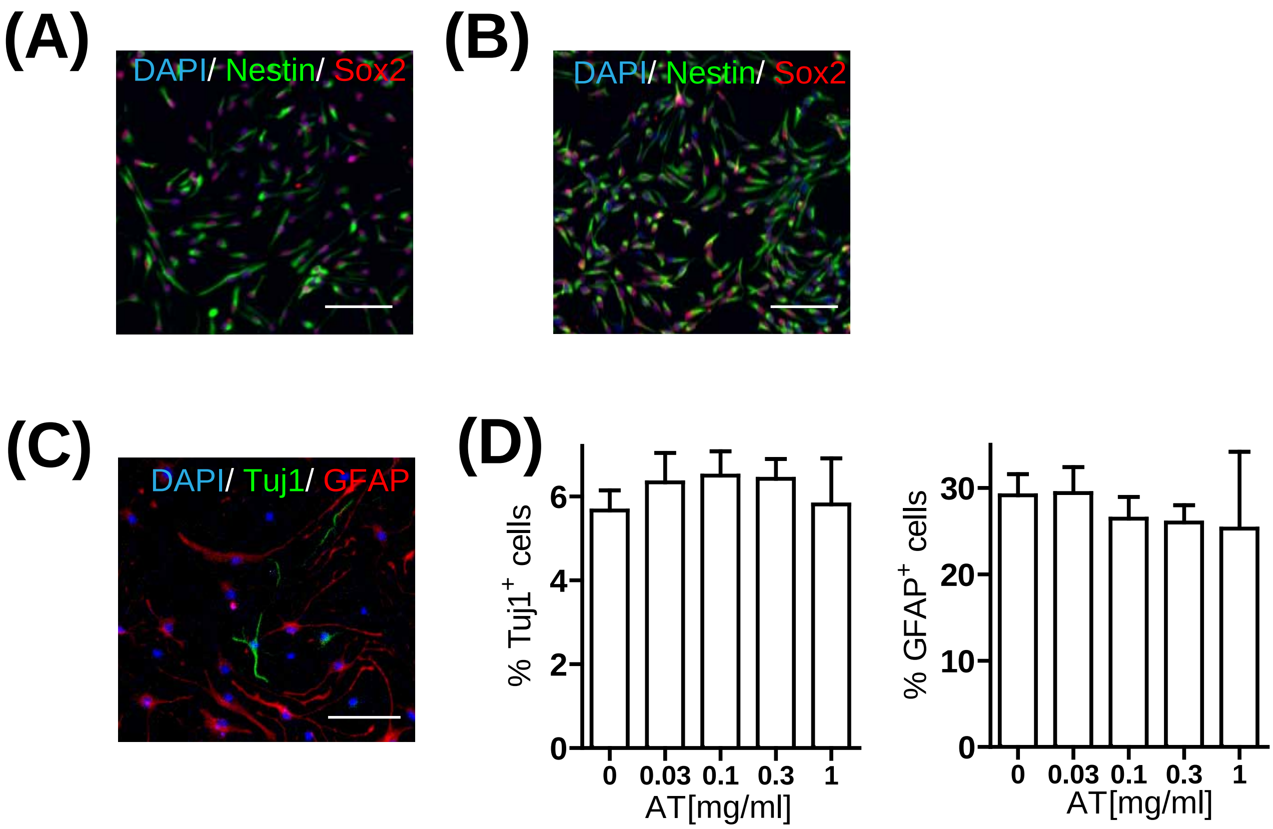

Fig. S2 Mao. et al.

Fig. S3 HPLC analysis of AT, ATE, ATB and ATW. The experiments were performed on a Waters 2695 system equipped with a UV detector. The profiles of (A) AT, (B) ATE, (C) ATB and (D) ATW were shown. Column: Cosmosil (C18, 5 μm, 250 × 4.6 mm); column temperature: 35 ℃; flow rate: 1.0 ml/min; mobile phase: MeOH (solvent A) and H2O (solvent B) with a linear gradient: 0-10 min (10-55%, A), 10-20 min (55-60%, A), 20-40 min (60-75%, A), 40-50 min (75-100%, A), 50-65 min (100%, A); UV detection at 208 nm; β-asarone, t_R = 25.0 min; α-asarone, t_R = 27.3 min.

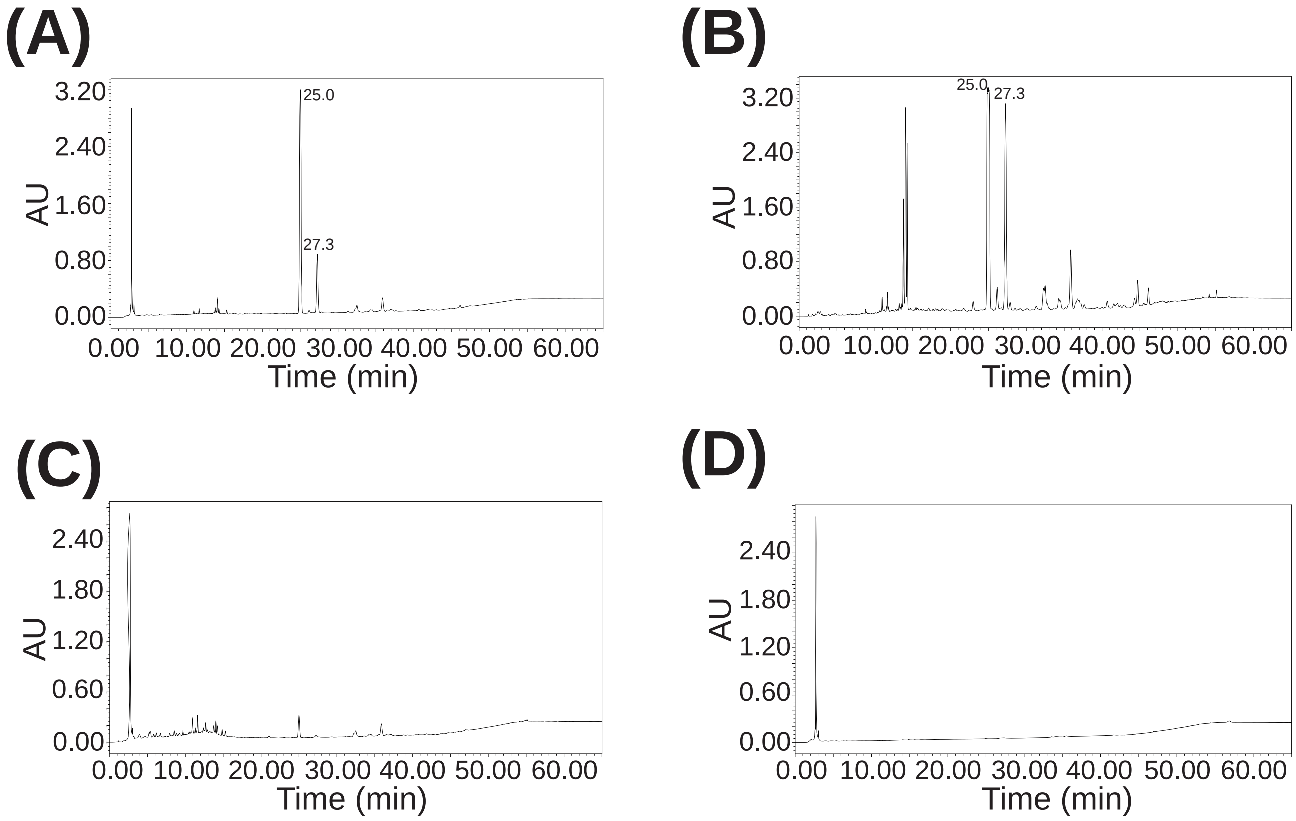

Fig. S3 Mao. et al.

Fig. S4 Effects of asarones on NPC differentiation and neurite outgrowth. (A) Monolayer embryonic neural precursor cultures were treated with the three fractions of different concentrations for 14 hours in the absence of EGF or bFGF. EdU was added 2 hours prior to fixation. EdU⁺ cells (as a percentage of total cells) in the culture were visualized and quantified. N = 8 independent experiments. (B) Percentage of EdU⁺ embryonic neural precursors treated with α-asarone, β-asarone and two analogues in ATE in the absence of growth factors. N = 8 independent experiments. (C) Percentage of Tuj1⁺ (left) or GFAP⁺ (right) cells in the adult hippocampal NPC cultures treated with asarones of different concentrations after 5 days under the differentiation condition. N = 5 independent experiments. (D-F) Primary neuron cultures were treated with AT or asarones from day 3 to day 7 in vitro followed by immunostaining for MAP2 (red; DAPI, blue) to visualize neurite outgrowth. (D) Representative images of MAP2 (red) staining and DAPI (blue) counterstain in the cultures treated with vehicle (ctrl), 10 μg/ml AT, 0.1 μM α-asarone or 0.1 μM β-asarone. (E) Quantification of neurite length of MAP2⁺ neurons. N = 4 independent experiments. (F) Quantification of neurite number per cell. N = 4 independent experiments. Quantifications are presented as mean ± SEM; *P < 0.05, **P < 0.01, ***P < 0.001, analyzed by one-way ANOVA followed by Fisher's protected least significant difference test; scale bars, 100 μm.

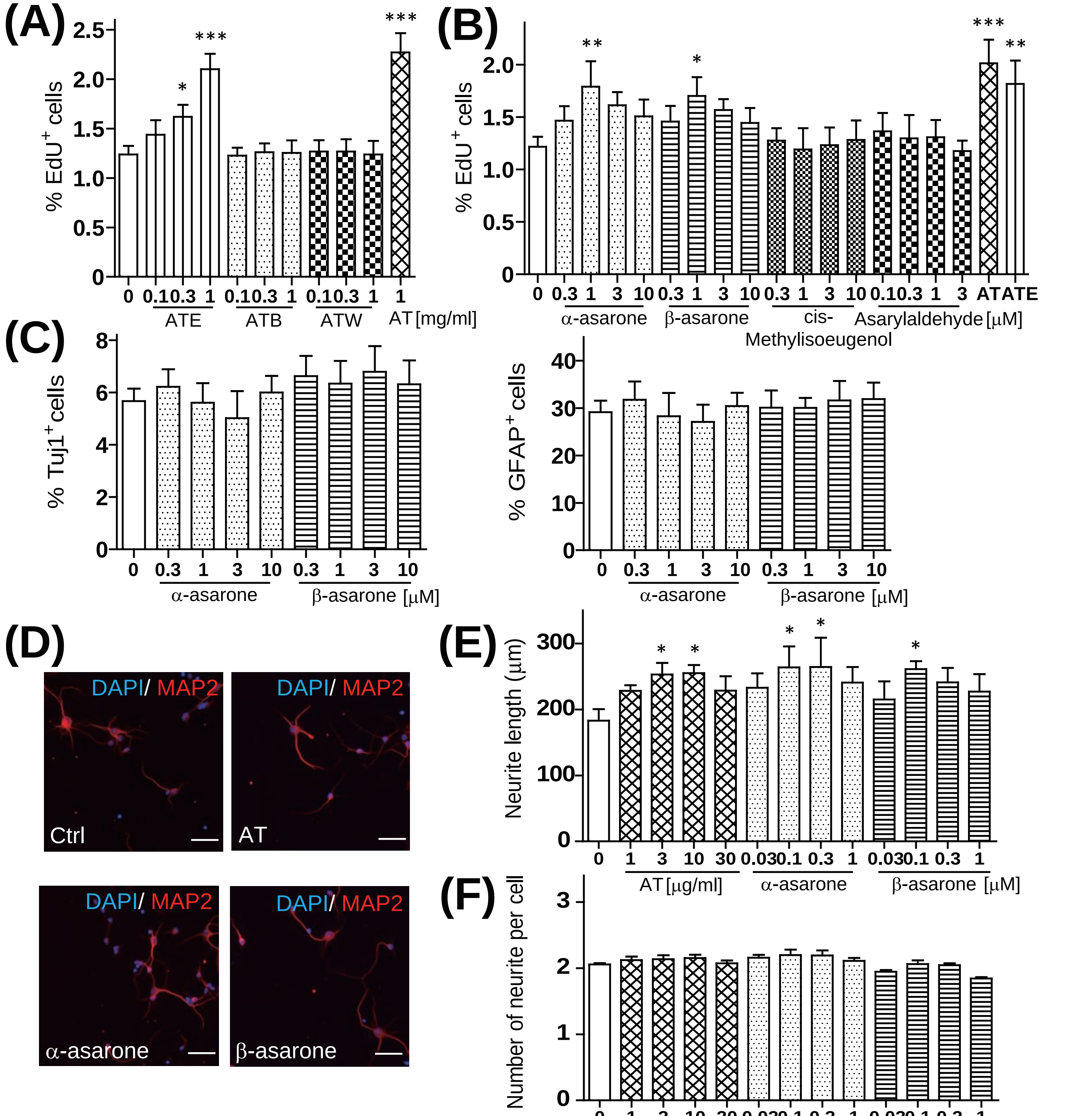

Fig. S4 Mao. et al.

Fig. S5 Effects of AT and asarones on neuroblastoma cell line proliferation. MTT analysis for proliferation of SH-SY5Y with different treatments in serum-free medium for 2 days. Quantifications are presented as mean ± SEM; ***$P < 0.001$; analyzed by one-way ANOVA followed by Fisher's protected least significant difference test.

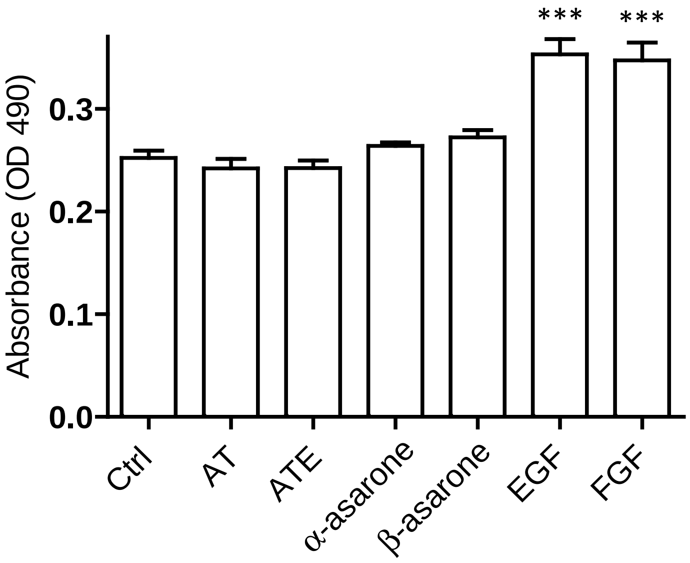

Fig. S5 Mao. et al.

Fig. S6 Effects of asarones on adult hippocampal neurogenesis. (A-C) The experiment was performed as depicted in Fig. 5A. The DG sections from the mice treated with (A) vehicle (Ctrl), (B) α-asarone and (C) β-asarone were immunostained for GFAP (red), Nestin (green) and BrdU (grey). Scale bars, 25 μm. (D) Quantification of $GFAP^{+}Nestin^{+}BrdU^{+}$ cells. N = 5 to 6 per group. (E-G) Tbr2 (green), BrdU (red) and DAPI (blue) co-staining of the DG sections from the mice treated with (E) vehicle (Ctrl), (F) α-asarone and (G) β-asarone. Scale bars, 100 μm. (H) Quantification of $Tbr2^{+}BrdU^{+}$ cells. N = 5 to 6 per group. (I-K) Ki67 (red), Dcx (green) and DAPI (blue) co-staining of the DG sections from the mice treated with (I) vehicle (Ctrl), (J) α-asarone and (K) β-asarone. Scale bars, 100 μm. (L-N) (L) $Ki67^{+}$ cells, (M) $Dcx^{+}Ki67^{+}$ cells and (N) $Dcx^{+}Ki67^{-}$ cells were quantified as indicated in (I-K). N = 5 to 6 per group. (O) Quantification of $Sox2^{+}$ cells in the DG sections from mice treated with vehicle (Ctrl) or asarones. N = 3 per group. (P) Quantification of Dcx and TUNEL double-stained cells in the DG sections from mice treated with vehicle (Ctrl) or asarones. N = 3 per group. (Q) Confocal images of c-Fos (blue), BrdU (red) and NeuN (green) co-staining in the DG from the mice treated with asarones. The experiment was performed as depicted in Fig. 5F. Scale bars, 10 μm. (R) The experiment was performed as depicted in Fig. 5F. The number of $GFAP^{+}BrdU^{+}$ cells of mice treated with vehicle (Ctrl) or asarones were determined. N = 4 to 5 per group. (S, T) Novel object recognition analysis. Preference scores of training phase (S) and Recognition Index of testing phase (T) across a one-day delay interval were shown, respectively. N = 5–8 for each group. Quantifications are presented as mean ± SEM; $^{*}P < 0.05$, $^{**}P < 0.01$, analyzed by one-way ANOVA followed by Fisher's protected least significant difference test; $^{\#}P < 0.05$, $^{\#\#}P < 0.01$, one-sample t test compared with 50% chance level.

Fig. S6 Mao. et al.

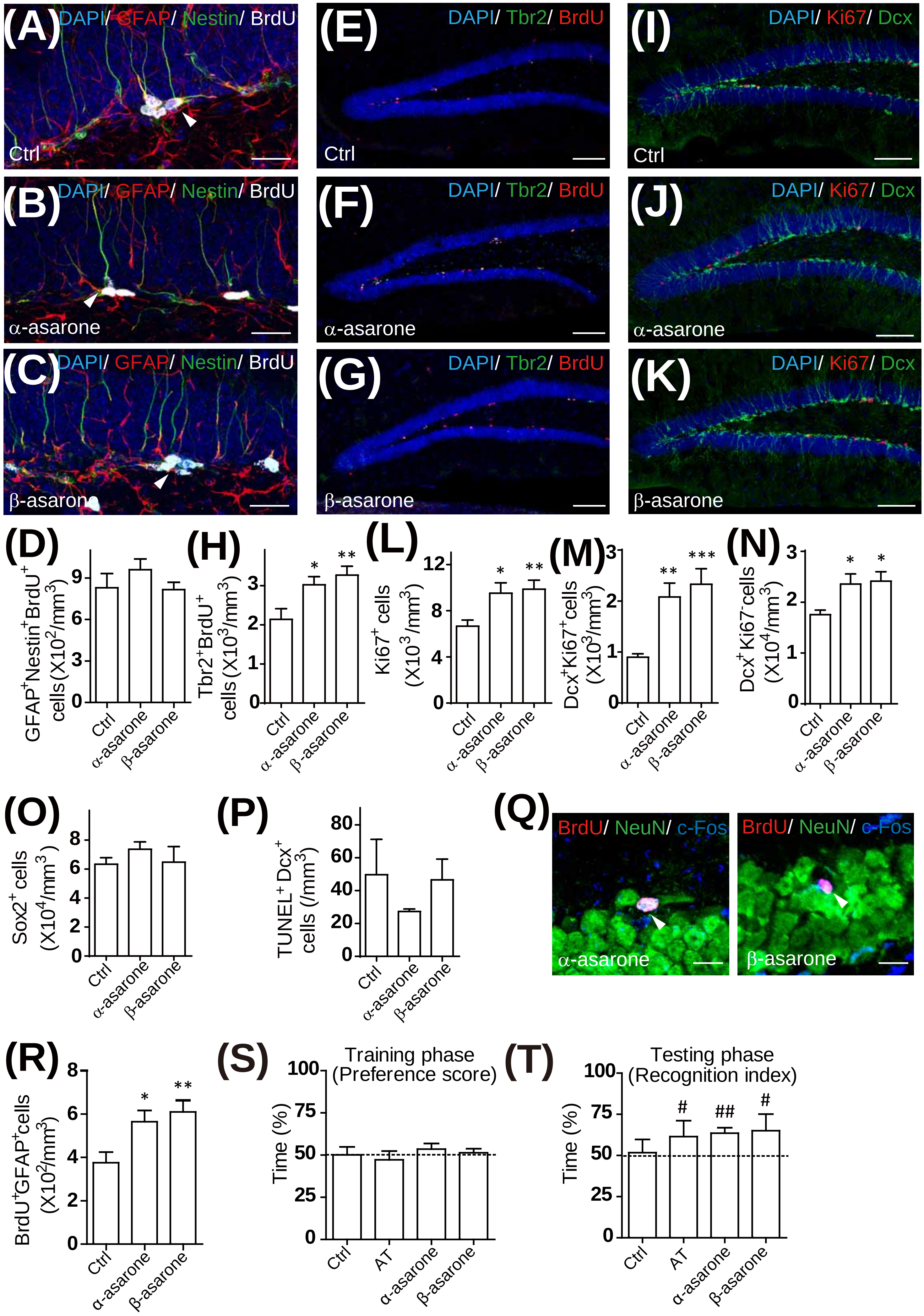

Fig. S6 Mao. et al.

Fig. S7 Short-term treatment with AT and asarones promotes hippocampal NPC proliferation. 11-week-old mice were treated with AT and asarones for 5 days and proliferating cells were labeled by BrdU injections. DG sections from mice treated with (A) vehicle (Ctrl), (B) AT, (C) α-asarone and (D) β-asarone were immunostained for BrdU and were quantified as in (E). N = 5–7 for each group. Quantifications are presented as mean ± SEM; *P < 0.05, **P < 0.01, analyzed by one-way ANOVA followed by Fisher's protected least significant difference test; scale bars, 100 μm.

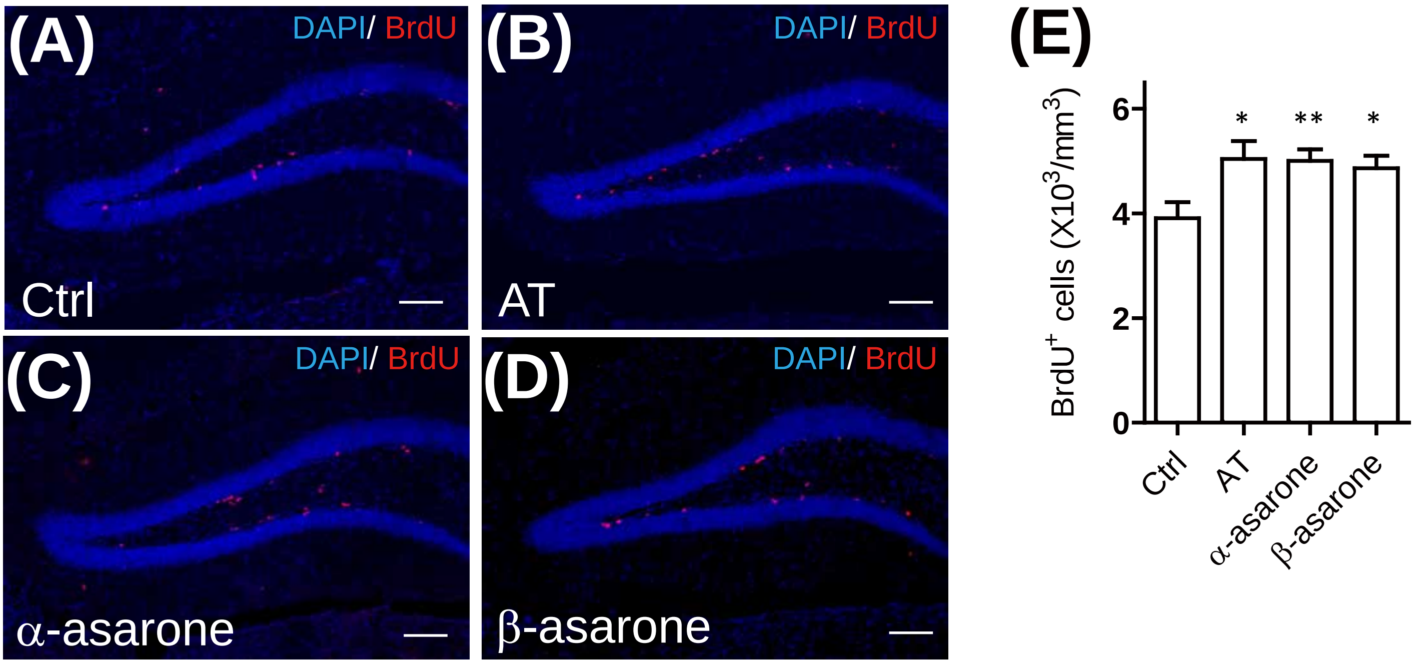

Fig. S7 Mao. et al.
